# The Silicone Rubber (SR) Composites: The Assessment of Changes in Hardness, Thermal Properties and Surface Quality Materials After Accelerated Aging Process

**DOI:** 10.3390/polym18131631

**Published:** 2026-06-30

**Authors:** Ewa Miękoś, Marek Zieliński, Dorota Czarnecka-Komorowska, Michał Cichomski, Tomasz Klepka, Dominika Drzewiecka, Dariusz Sroczyński, Anna Fenyk

**Affiliations:** 1 Department of Inorganic and Analytical Chemistry, Faculty of Chemistry, University of Lodz, Tamka 12, 91-403 Lodz, Poland; marek.zielinski@chemia.uni.lodz.pl (M.Z.); dariusz.sroczynski@chemia.uni.lodz.pl (D.S.); anna.fenyk@chemia.uni.lodz.pl (A.F.); 2 Department of Plastics Division, Institute of Materials Technology, Poznan University of Technology, Piotrowo 3, 61-136 Poznan, Poland; dorota.czarnecka-komorowska@put.poznan.pl; 3 Department of Materials Technology and Chemistry, Faculty of Chemistry, University of Lodz, Pomorska 163, 90-236 Lodz, Poland; michal.cichomski@chemia.uni.lodz.pl; 4 Department of Technology and Polymer Processing, Faculty of Mechanical Engineering, Lublin University of Technology, Nadbystrzycka 36, 20-618 Lublin, Poland; t.klepka@pollub.pl; 5 Department of Biology of Bacteria, Faculty of Biology and Environmental Protection, University of Lodz, Banacha 12/16, 90-237 Lodz, Poland; dominika.drzewiecka@biol.uni.lodz.pl

**Keywords:** silicone rubber composites, constant magnetic field, plant-based fillers, *Curcuma longa*, *Solidago virgaurea*, carbonyl iron

## Abstract

In this paper, the accelerated aging process of a layer of polymer composites based on the silicone rubber modified by plant-based fillers and iron carbonyl was investigated. The color change, Shore’s hardness, thermal resistance, and surface quality of the composites after weathering were tested. The degradation behavior of the silicone rubber and its composites was characterized by TGA and FTIR techniques, as well as optical microscopy. It was found that the aging process increased the hardness of the silicone rubber composites by approx. 10% compared to pure silicone, and by a further 10% by weight in the case of composites containing plant-based fillers, such as turmeric and goldenrod. The promising results indicate that a SR/CLP/Fe/AM composite modified with carbonyl iron and a surfactant acts as an effective stabilizer for the silicone rubber matrix. An increase in the maximum temperature of 40 °C was also recorded compared to the unmodified silicone rubber. The results showed that the surface color of the aged samples changed in terms of lightness and total color. It was found that silicone rubber composites containing plant-based fillers exhibit properties characteristic of age-resistant materials; in particular, the SR/CLP/Fe composites modified with carbonyl iron are more resistant to water and light during accelerated aging than unmodified silicone rubber, which ensures a longer service life.

## 1. Introduction

Nowadays, polymeric materials, including silicone rubber, are used in many outdoor applications, such as in the construction and automotive industries. These products must endure specific weather conditions, which place high demands on their durability, color retention, and thermal stability throughout their service life. One way of shaping such characteristics and structure is through polymer modification, which allows the production of polymeric materials that are more resistant to degradation processes.

The silicone rubber is a synthetic elastomer based on inorganic silicon, consisting mainly of polysiloxanes, oxygen, and organic groups (carbon, hydrogen). Its structure is based on silicon-oxygen chains. The Si–O bond of silicone rubber gives, in terms of heat resistance, chemical stability, electrical insulating, abrasion resistance, weatherability, and ozone resistance. With these unique characteristics, silicone rubber and composites of silicone rubber have been widely used in various industries like the munitions industry, aerospace, automobile (gaskets), construction, electronics (fuel cells), and the medical industry [1]. They are low-cost materials that can be processed using simple manufacturing techniques. Due to the wide range of applications for silicone rubber in various environmental conditions, it is essential to understand its resistance to environmental factors that cause its degradation [2,3]. In the presence of water, at temperatures above 120 °C, silicone rubber undergoes hydrolytic degradation, resulting in the breakdown of the main chain [2]. In contrast, the thermal aging of silicone rubber in the presence of oxygen causes cross-linking, resulting from a combination of increased molecular energy and the loss of methyl groups, which leads to the formation of silanol groups that undergo condensation to form new Si-O-Si cross-links [4].

Degradation processes caused by aging of the polymer material by means of photoirradiation, thermal degradation, photooxidation, as well as through hydrolysis processes, lead to a number of changes in the physical, chemical, and mechanical properties of polymer composites. Aging of polymers or their composites is the sum of irreversible changes in the material properties occurring as a result of exposure to light, sun, oxygen, radiation, and heat. These multiple factors often work together, and therefore, it is important to know the properties of plastics in different degradation environments [5,6].

Aging of layered composites has a different impact on the physicochemical behavior of the material, manifested by changes in its mechanical and thermal properties. In some works, the authors used glass fibers to modify and strengthen the created composites [7]. To improve resistance to UV and hydrolytic degradation, polymers are physically modified by adding stabilizers and antiaging agents, such as carbon black, MMT, glass fiber, fly ash, zeolite, cellulose, etc. [8,9,10,11,12].

Given the significance of this issue, many researchers are conducting studies focused on assessing the impact of time, temperature, and environmental conditions on the rate of degradation of polymers, including silicone-reinforced composites [13,14,15,16]. For example, Feng et al. [15] investigated the degradation of silicone rubbers with different hardness in various aqueous solutions. They found that silicone rubbers became more durable in aqueous solutions with the increase in hardness. Li et al. [16] investigated the degradation of silicone rubbers exposed to simulated and accelerated proton exchange membrane fuel cell environments. The result showed that silicone rubbers degraded more severely with increasing acid and temperature. The degradation of silicone rubbers was attributed to chemical decomposition of the silicon-based backbone, accompanied by leaching of fillers.

Multilayer composites, which are used in many industries, are also subject to aging processes. Zubair et al. [8] developed three different one-layer, two-layer, and three-layer composites using only fiberglass, while the resin material induced the shape memory function. They concluded that increasing the number of layers of glass fiber fabric reduced the durability of the shape due to the increase in stiffness; however, changing the characterization temperature improved the shape stability of the three-layer composites.

Garbacz et al. [9] presented studies of polypropylene blends with three types of mineral fillers, such as aluminosilicates (zeolite), fly ash, and gypsum powder derived from post-production waste. Aging of the tested composites within the 120–360 h range causes a rapid decrease in the resistance of the composite to impact. The authors emphasized that the change in the hardness of zeolite and fly ash is related to the type of polymer used and the technological conditions of the process, and not to the type of mineral fillers. Samujło et al. [11] determined the changes in the selected mechanical properties and flammability of polypropylene modified with natural fillers (sunflower husk, wheat bran, pine sawdust, and talc) caused by aging. A beneficial effect was a relatively small influence of aging on the mechanical properties of the modified polypropylene fillers, which is a good prognosis for the service life of products made of this type of material. The study by Sánchez et al. [17] aimed to evaluate the effect of thermal aging on the mechanical properties of natural rubber-based compounds reinforced with carbon soot, which were exposed to thermal oxidation. Szymiczek et al. [18] investigated the effect of aging in water at a temperature of 100 °C on selected mechanical properties of polypropylene with organic fillers (hemp chaff, oak, birch, and maple leaf mixture). They found that the higher the filler content, the lower the tensile strength and elongation at break, and the lower the impact strength at each stage of aging. Moisture content in polymer composites often causes degradation by swelling and hydrolysis; faster degradation can be caused by water absorption than in any other environment [19]. The work of Magiera et al. [14] investigated the effect of fillers, including fly ash, on the chemical structure, mechanical properties, and aging of rigid composite polyurethane foam materials. The aging process resulted in deterioration of mechanical performance, and exposure to ambient conditions had a more pronounced effect on mechanical parameters compared to accelerated aging under laboratory conditions. In the paper [13], the authors conducted thermal aging experiments to determine its effect on the mechanical properties of thermoplastic polyurethane (TPU). As a result of the tests, the authors observed not only changes in the appearance of TPU and weight loss, but also an improvement in TPU stiffness as a result of thermal aging.

Bouregba et al. [20] analyzed the consequences of thermal degradation on the thermal and mechanical properties of three separate materials: polypropylene (PP), polypropylene/talc, and polypropylene/carbon soot (PP/PCS). The obtained results indicated that reinforcement of polypropylene with mineral particles generally improves its thermal stability, crystallinity, and mechanical properties. They found that the carbon soot showed better thermal stability compared to talc before aging. Susanto et al. [21] in their publication used a new reinforcing filler in the form of modified starch in natural rubber (NR) and styrene-butadiene rubber (SBR) composites. The results showed that this filler could be used due to its ability to slightly improve the composite hardness, tensile strength, and tear strength, and the duration of thermo-oxidative aging affected the alteration of these properties. In the paper of Szatkowski et al. [22], polylactide-based composites with plant-based fillers were prepared: microcellulose powder, short flax fibers, and wood flour. The properties of the poly(lactic acid) (PLA)-based biocomposite were characterized in terms of mechanical and surface properties before and after the ultraviolet (UV) aging process to determine the effect of each cellulose-based additive on the UV-induced degradation process. They found that the addition of cellulose improves the UV aging properties of the material, but the form of the selected cellulose plays a key role in this case. Bendjaouahdou et al. [23] investigated the effects of thermal and UV aging on a blend of natural rubber (NR) SMR-ω and polypropylene. They found that the results obtained from thermal and UV aging demonstrated that the mixture containing 30% by weight of natural rubber is more resistant to heat and UV radiation than other tested blend compositions. The composite was modified by adding 10% by weight of plant-based fillers, such as goldenrod (*Solidago virgaurea* L.) and turmeric *(Curcuma longa* L.), and magnetic particles [24] with ferromagnetic properties in the form of carbonyl iron (Fe) in the amount of 20 wt.% [25]. Common goldenrod (*Solidago virgaurea* L.) is a plant commonly found in many countries across Europe, North America, and Asia. It grows in forests, meadows, and on sandy wasteland. It flowers from July to September (yellow inflorescences) and is used as a herbal remedy for kidney stones and urinary tract infections; it can be used to treat gastritis and enteritis, lower blood pressure, and aid in the treatment of atherosclerosis. It contains phenolic glycosides, phenolic acids, and flavonoids. It has antiseptic, antibacterial, and astringent properties and promotes wound healing. It was chosen as a filler because it is abundant, can be easily processed for use as a filler (drying, grinding), is biodegradable, and also has medicinal properties. Turmeric (*Curcuma longa* L.) is a perennial plant 60–100 cm tall, cultivated in China, India, Thailand, and the Philippines [26]. It may contain around 200 medicinal compounds. It is soluble in fats and alcohol, but not in water. It has anti-cancer properties, reduces inflammation of the bile ducts, neutralizes free radicals, and protects against so-called oxidative stress. It is also notable for its bactericidal properties. It enhances the effectiveness of antibiotics in destroying pathogenic bacteria. It was chosen as a filler because it is biodegradable and also possesses medicinal properties. It is one of the most promising plants for combating various viruses. Magnetic particles (carbonyl iron) improve the thermal stability of silicone rubber by acting as a highly reactive radical acceptor and a cross-linking catalyst [27]. This increases the material’s resistance to degradation at high temperatures. In addition, the iron particles act as physical cross-linking nodes, which stiffen the material and reinforce the matrix, forming a framework that restricts the movement of polymer molecules. This results in an immediate increase in the elastic modulus, making the materials harder. Consequently, the tensile strength, Young’s modulus, and thermal conductivity of the composite are increased.

The work of Abshinova et al. [28] investigated the frequency-temperature dependence of the complex permeability and permittivity of polydimethylsiloxane (PDMS) filled with carbonyl iron (CI) powders. Englert et al. [29] focused on thermal oxidative stabilization of silicone rubber, which is achieved by titanium oxide-based fillers.

In this paper, the effect of accelerated aging on the hardness, thermal stability, and functional properties, such as color and surface quality, of a layer of silicone rubber-based polymer composites was investigated. The developed compositions have a wide range of applications, including in the furniture industry as flexible door-handle covers, in the construction industry as window and door seals, and as washers and gaskets in the automotive and electronics industries. For this reason, research was carried out with the aim of developing new, environmentally friendly multilayer composites based on silicone rubber with specific properties, characterized by good resistance to environmental factors in difficult conditions.

## 2. Materials and Methods

### 2.1. Polymer Composites

**Figure 1:**
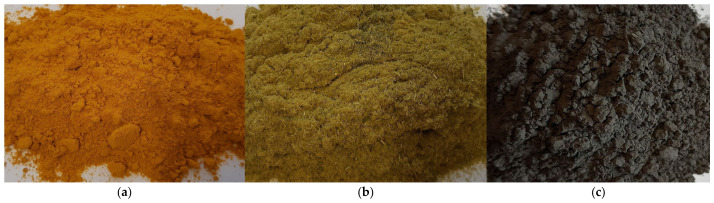
The fillers used for the modification of silicone rubber: (**a**) turmeric (*Curcuma longa* L.), (**b**) goldenrod (*Solidago virgaurea* L.), (**c**) carbonyl iron (Fe).

The two-layer polymer composites were prepared using the following components: a silicone rubber with the trade name Polastosil M-56 [30], a material with lower elasticity (the down layer), and a silicone rubber with the trade name Gumosil B, a material with higher elasticity (the upper layer). The OL-1 catalyst was used as the curing agent. The Polastosil M-56, Gumosil B, and OL-1 catalyst were manufactured by Chemical Plant Silikony Polskie Sp. z o.o. of Nowa Sarzyna, Poland. The composite was modified by adding 10% by weight of plant-based fillers (Figure 1a,b), such as goldenrod (*Solidago virgaurea* L.) with disinfectant and antibacterial properties and turmeric (*Curcuma longa* L.), with anti-cancer, bactericidal, and antiviral properties. The plant-based fillers were produced by Dary Podlasia, Bielsk Podlaski (Poland). Turmeric had a particle size ranging from approx. 20 μm to over 300 μm. Following drying in a dryer (at 50–60 °C for approx. 6–12 h) and grinding, the moisture content ranged from 7% to 14%. The turmeric was 100 percent pure and originated from Poland. Goldenrod had an average particle size of approximately 47–50 μm for 90% of the particles and approximately 126–138 μm for 10%. Following drying at a temperature of approx. 40 °C, the moisture content was about 2%. The purity of common goldenrod was 100%, and it was sourced from Poland. Pharmaceutical goldenrod guarantees a specific content of active substances (e.g., flavonoids such as rutin and quercetin). In the case of field goldenrod, the amount of active substances may vary depending on weather and soil conditions.

The top layer of some two-layer polymer composites with silicone rubber (SR) matrix was modified by magnetic particles with ferromagnetic properties in the form of carbonyl iron (Fe) (Figure 1c) in the amount of 20 wt.%. The carbonyl iron was derived from the decomposition of iron pentacarbonyl at high temperature (97%, Alfa Aesar, Thermo Fisher Scientific, Heysham, Lancashire, UK). Carbonyl iron had a particle size of 5–6.5 μm. One of the SR composites, the SR/CLP/Fe/AM, contained an additional amphoteric surfactant named ROKAmina L30 MB (*Lauryl betaine*) [31] produced by PCC Exol S.A. (Brzeg Dolny, Poland) The raw material included in this surfactant is a derivative of palm kernel oil from certified Guinean oil plantations [31]. Due to its structure, it had the ability to reduce the surface tension at the interface of the phases of the liquid system, thus changing its free energy.

### 2.2. Preparation of SR Silicone Composites Test Samples

**Figure 2:**
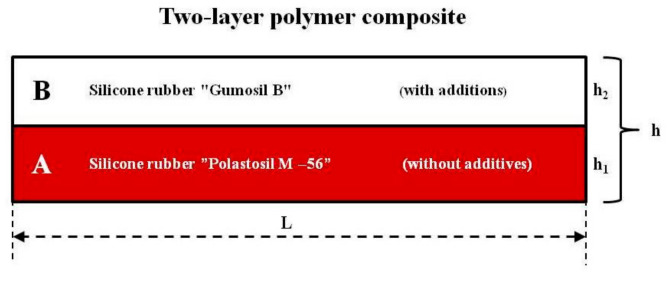
A two-layer polymer composite with a length (L), total thickness (h), which consisted of layers (**A**) of thickness (h_1_) and (**B**) of thickness (h_2_).

All the tested samples were prepared according to the same procedure. The bottom layer of the composite (A), with a thickness of h_1_, was made of the Polastosil M-56 type silicone rubber, with lower elasticity, containing no additives. This layer was the substrate of the two-layer composite, as shown in Figure 2.

The content of each component in the composite was calculated in relation to the mass of the modified layer, i.e., layer B, based on ‘Gumosil B’ silicone rubber.

After developing the composition of the upper layer of the composite (B) with an h_2_ thickness, containing admixtures, the individual components were weighed in the following order: Gumosil B type silicone rubber (with greater flexibility), plant admixture of goldenrod (*Solidago virgaurea* L.) or turmeric (*Curcuma longa* L.), in the amount of 10% by weight; for selected samples, magnetic particles in the form of carbonyl iron (Fe) in the amount of 20% by weight and the OL-1 hardener for silicone rubber in the amount of 3% in relation to the amount of the polymer were added. The whole was mixed with the Rod Mechanical Stirrer SE-100 (Beriain, Spain) at a speed of 300 rpm for 180 s. The mixed liquid mass of the sample was placed in molds prepared according to the PN-EN ISO 10210:2018-1 standard. The test specimens had the following dimensions: length (L) = 120 mm, height (h) = 10 mm (i.e., h_1_ and h_2_ were each 5 mm), and width (w) = 10 mm. No delamination of the composite occurred following the tests.

The layer B composition of the tested samples is presented in Table 1.

### 2.3. Accelerated Aging Method of SR Composites

Accelerated aging measurements were carried out in a chamber of AN 154 866 XENTEST 2200 ADVANCED type, with a Xenon arc-lamps and with a wet phase (all from Agencja Anticorr Gdańsk Sp. z o.o., Gdańsk, Poland). The tests were carried out in accordance with the PN-EN ISO 4892-2 standard [32]. The criterion for ending the aging process was the dose of the emitted radiation of 3.6 GJ at 1000 kWh.

The SR composites were exposed to the light of a xenon arc lamp, characterized by white light similar to sunlight, in the presence of moisture, in order to reproduce the effects of weather conditions that occur in the environment of actual use of the material, when exposed to daylight. The samples were subjected to the lamp light under controlled conditions, such as time, temperature, humidity, and irradiance. The exposure conditions (artificial weather conditions), corresponding to the actual conditions during 1 year, were as follows: time 102 min dry, 18 min water spraying; the irradiance at 340 nm wavelength 0.51 nm (W/m^2^); black pattern temperature: 65 ± 3 °C; chamber temperature: 38 ± 3 °C; relative humidity: 50% ± 10.

### 2.4. Testing Methods Characteristics

#### 2.4.1. Shore’s Hardness Analysis

The hardness of samples was measured using a Shore hardness tester (Mitutoyo CTS-103, Mitutoyo Corporation, Kanagawa, Japan) according to the ISO 868:2005 standard [33]. The hardness was indicative of an average penetration value (Shore degrees on the A scale) based on 5 readings from tests.

#### 2.4.2. TGA Analysis

The thermogravimetric (TGA) method, according to the ISO 11358-1:2022 [34] was performed using a TG 209 F1 Libra apparatus (Netzsch, Ltd., Selb, Germany) operating in a protective nitrogen atmosphere with a 60 mL/min flow rate. The samples (9.0 ± 1.0 mg) were placed in a ceramic crucible and heated from room temperature to 900 °C at a rate of 10 °C/min.

#### 2.4.3. Color Analysis

According to the ISO 4582:2025 standard [35], color measurements were performed to determine the color change in the composites after accelerated aging. The color of the samples was determined using the CIE 1976 L* a* b* system (CIE Lab) [36] on a rectangular coordinate system. All color functions were calculated for a D65 light source and a 2° angle observer using tristimulus values obtained with an EnviSense NR145 colorimeter (EnviSense dr Barbara Mirosław, Lublin, Poland) with measurement geometry 45°/0° for the assessment of the color standard.

The measurement area was 8 mm in diameter, 0° viewing geometry (d/0°). The evaluation and quantification of the sample’s color appearance were analyzed based on chrominance coordinates a* and b*, along with brightness L*, as variables to quantify material deterioration. Hue and color saturation are on the a* and b* axes, where a is the axis of the red-green character (+a-redder; −a-greener); b is the axis of the yellow-blue character (+b = yellower; −b = bluer) [37]. L is the lightness (luminance) parameter (a maximum value of 100 represents a perfectly reflecting diffuser; the minimum value of zero represents the color black). On the other hand, (ΔL*) expresses the difference in brightness between the standard and the aged sample. The sign of this difference determines the direction of the change in brightness: a negative sign indicates a change to darker, while a positive sign indicates a change to lighter.

The color change (Δ*E*) was calculated using the Euclidean formula Equation (1). As follows (1), the values measured for unaged samples were used as a standard for calculations [36,37].

(1) $$∆E\;=\sqrt{{\left(∆a\right)}^{2}+{\left(∆b\right)}^{2}+{\left(∆L\right)}^{2}}$$

where $∆a=a_{0}-a_{UV};$ $∆b=b_{0}-b_{UV};$ $∆L=L_{0}-L_{UV}$.

#### 2.4.4. Optical Microscopy (OM) Analysis

In order to compare changes in the color of pure silicone rubber and its composites containing carbonyl iron and plant-based fillers, such as turmeric and goldenrod, following the aging process, microscopic photographs of the sample surfaces were taken. Microphotographs of the samples were taken at 20× magnification using an Opta-tech SM series polarizing optical microscope (OPTA-TECH, Warsaw, Poland). The photographs of the samples were taken using an Opta-tech HDMI digital camera, which is compatible with the microscope.

#### 2.4.5. FTIR Analysis

Fourier transform infrared (FTIR) spectroscopy was performed using the Jasco FT/IR-4600 apparatus (JASCO International Co., Ltd., Tokyo, Japan) equipped for working in attenuated total reflectance (ATR) mode. FTIR analyses were carried out using 64 scans at a resolution of 4 cm^−1^ in the wavenumber range of 4000–400 cm^−1^.

#### 2.4.6. Statistical Analysis

The results shown in the graphs are the arithmetic mean calculated from five measurements. Their standard deviations were calculated with the STDEV.S function from Excel 365 (Microsoft Corporation, Redmond, WA, USA) and were presented in the graphs as error bars. The statistical significance of the aging process on Shore hardness on the A scale, the absolute color difference (ΔE*), and the difference in brightness between the standard and the aged sample (ΔL*) of pure silicone rubber and its composites was demonstrated using the T.TEST function from Excel 365. This function calculates the *p*-value of Student’s *t*-test, allowing the determination of whether the difference between the means of two data sets is statistically significant or random. These calculations were performed at the significance level α = 0.05 as a two-tailed test since it is mathematically more conservative and evaluates the possibility of a significant change in both directions, and for paired data since a given sample was measured before and after the aging process. To demonstrate a statistically significant impact of the aging process on Shore hardness on the A scale of pure silicone rubber and its composite, the T.TEST function from Excel 365 was used. The calculated *p*-value of Student’s *t*-test was equal to 0.00045 and was much lower than the assumed significance threshold of α = 0.05. Thus, we conclude that the aging process has a statistically significant impact on the hardness of pure silicone rubber and its composites. To demonstrate a statistically significant impact of the aging process on the absolute color difference and the difference in brightness between the standard and the aged sample ΔL* of pure silicone rubber and its composite, the T.TEST function from Excel 365 was used. The calculated *p*-values of Student’s *t*-test were equal to 0.204 and 0.301, respectively, and were higher than the assumed significance threshold of α = 0.05. Thus, we conclude that the aging process does not have a statistically significant impact on the absolute color difference (ΔE*) and the difference in brightness between the standard and the aged sample (ΔL*) of pure silicone rubber and its composites.

## 3. Results and Discussion

### 3.1. Hardness of Silicone Rubber (SR) Composites Analysis

**Figure 3:**
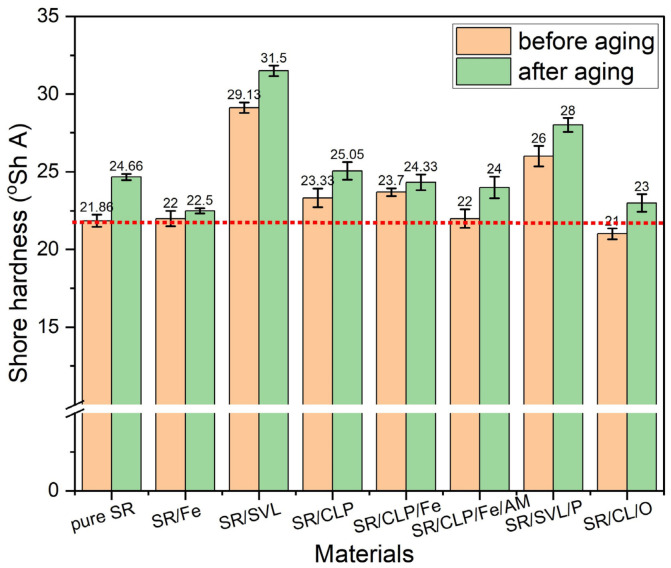
Shore hardness of pure silicone rubber (SR) and its composites before and after aging. The dashed line refers to the Shore hardness of pure silicone rubber (SR).

An analysis of changes in the material properties of silicone rubber-based composites under the influence of water and radiation was carried out on the basis of surface hardness tests on the samples. The pure silicone rubber (SR) and its composite with added fillers were subjected to Shore hardness on the A scale (in ^o^ShA) before and after the aging process. The results of the hardness tests are shown in Figure 3.

The results show that the hardness of silicone rubber-based composites modified with natural fillers and carbonyl iron is generally higher than that of unmodified (pure SR) silicone rubber, which is due to the addition of fillers to the polymer matrix. The SR/SVL composite with added turmeric exhibits a significant hardness of approx. 40%. The hardness results show that all composite samples subjected to the aging process exhibit higher hardness compared to the non-aged samples. The greatest change was observed in the SR composite modified by a curcuma filler (SR/SVL and SR/SVL/P), whose hardness increased by approx. 30% compared to the non-aged silicone rubber (pure SR).

The accelerated aging process increased the hardness of the pure silicone rubber by approx. 13%. The addition of plant-based fillers to silicone rubber caused an increase in the hardness of the composites (4, 5, 6, 8) by approx. 10%. The addition of carbonyl iron filler increased the hardness of the composites (2, 5, 6) compared to the pure SR by approx. 9%. The addition of the ROKAmina, amphoteric surfactant, to composite (6) resulted in a similar effect in an increase in hardness (6) by approx. 10%. The hardness of the sample’s surface after aging was increased because the polymer chains could not explore different configurations and behavior as less flexible, with the result that the material became more cross-linked [38]. A similar effect for silicone rubber (HTV—High Temperature Vulcanized) filled with polydimethylsiloxane was observed by Delor et al. [39]. On the other hand, the lower hardness of the SR modified by curcuma in oil, such as SR/CL/O, suggests that the plant-filler in oil has plasticized the silicone rubber. Hence, the composites are more flexible.

The observed changes in hardness indicate that composites containing natural fillers and modified with carbonyl iron, particularly the SP/CLP/Fe composite, exhibit greater resistance to water and light during accelerated aging than unmodified silicone rubber.

### 3.2. Thermal-Oxidative Degradation of the Silicone Rubber (SR) Composites Analysis

The thermal degradation behavior of pure silicone rubber (SR) and its composites was studied by TGA in a nitrogen atmosphere. The TGA method allows for the inference of the magnitude of thermal transformation as well as the temperature at which the transformation occurs. At this temperature, the mass of the tested material changes under the influence of heating and in a specific atmosphere. The mass changes can be determined by integrating the corresponding peak of the first derivative of the TGA curve to obtain the DTG curve. The thermal properties of the studied polymer composites were determined on the basis of TGA measurements and differential thermogravimetry (DTG), as the temperature at which the mass loss was 5% (T_5%_), 50% (T_50%_), and the temperature for peak degradation (T_max_). Thermogravimetric measurements are typically used to detect the composition of polymer mixtures and characterize the degradation process. In our case, the T_5%_ temperature of all samples, before and after the aging process, T_50%_, the maximum decomposition temperature T_max_, and residues at 900 °C are taken into account.

**Figure 4:**
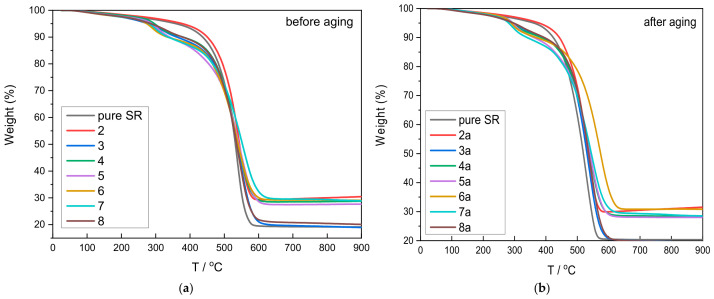
TGA curves of pure silicone rubber (SR) and its composites: (**a**) before and (**b**) after aging in nitrogen.

**Figure 5:**
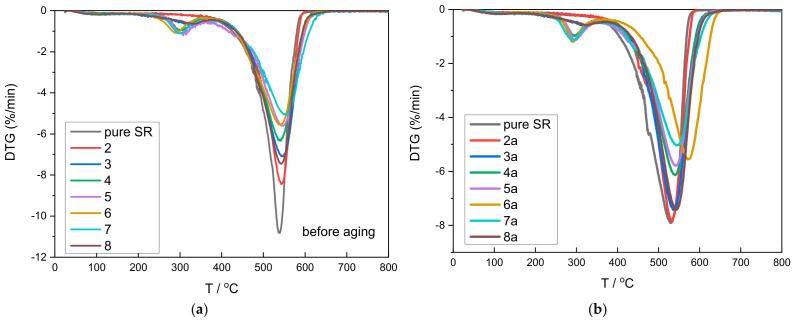
DTG curves of pure silicone rubber (SB) and its composites: (**a**) before and (**b**) after aging in nitrogen.

The TGA results are demonstrated in Figure 4 and Figure 5, which show the curves of mass loss (TG) and their derivatives (DTG) as a function of temperature. The characteristics data results of the TGA analysis performed for samples before and after aging are listed in Figure 4 and Figure 5 and in Table 2.

Detailed data on thermal parameters, including the temperature corresponding to a 5% mass loss (T_5%_), and silicone rubber SR degradation temperature (T_max_), is described by Zeng et al. [40].

Overall, the TGA results show that the onset temperature of polymer decomposition (T_5%_) for the SR/Fe composite containing carbonyl iron is the highest (approx. 380 °C) compared to unmodified silicone rubber. The onset temperature of silicone rubber decomposition is 359 °C [38]. This demonstrates the composite’s superior thermal stability. Therefore, the metal oxides such as Al_2_O_3_ and Fe_2_O_3_, and carbon nanomaterials, e.g., carbon black, carbon nanotubes, and graphene, used as additives, increase the high-temperature resistance of silicone rubber composites [41]. For the other composites containing natural fillers, such as goldenrod and turmeric, based on silicone rubber, a significant decrease in temperature was observed; the decomposition of the plant-based fillers, including goldenrod biomass, begins at a temperature of 200–250 °C [42].

When analyzing the effect of the aging process on the behavior of composites, it can be observed that composites modified with Fe carbonyl and ROKAmine exhibit a marked shift in the 5% degradation onset temperature (T_5%_) towards higher values compared to unaged composites. The highest increase, of approximately 15%, was recorded for sample (6a) modified with Fe and ROKAmine. This demonstrates that the addition of fillers significantly improves the thermal properties of the compositions, indicating that the filler has an antiaging effect and that the resulting composition is more resistant to the thermal degradation of silicone rubber.

For the first correlation (Table 2), three samples were taken into account before and after accelerated aging. These were samples 1 and 1a, containing silicone rubber only, samples 3 and 3a, containing silicone rubber with goldenrod (*Solidago virgaurea*), and samples 8 and 8a, containing silicone rubber with turmeric *Curcuma longa* L. (oil). The thermal decomposition of 5% of the composite mass and 50% of the composite mass occurs much earlier for the composites with plant admixtures.

The SR/CLP/Fe/AM (6) sample contains a surfactant, a surface-active compound called ROKAmina L30B, which reduces the surface tension of the solution prior to polymerization. It is a liquid at 20–25 °C, with a pH of 7 in a 10% solution. It contains an admixture of monochloroacetic acid (10 ppm) and dichloroacetic acid (29 ppm). Surfactants in silicone rubber systems act as interfacial modifiers. They ensure the uniform dispersion of the fillers and additives used, preventing their agglomeration, which increases the mechanical strength of the new material; they also affect the polymer’s slip properties and influence its hardness and texture. By reducing surface tension, the surfactant improves the adhesion of silicone rubber to other materials, facilitates mold casting, and prevents delamination in multi-component systems. The formation of micelles by surfactant molecules determines the ability of surfactants to solubilize hydrophobic substances, including lipid vesicles. The term ‘solubilization’ should be understood as the spontaneous absorption of hydrophobic substances by micellar aggregates of a surfactant, with the aim of forming a thermodynamically stable solution characterized by reduced thermodynamic activity. The solubilization process is particularly important when seeking to increase the solubility of compounds that are naturally insoluble in a given environment. Surfactants also act as special coatings for nanoparticles, improving their flexibility and their ability to distribute molecules of various substances.

**Figure 6:**
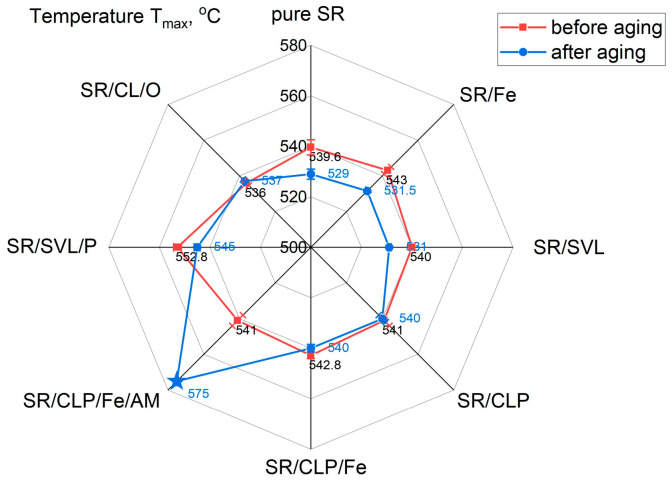
Comparison of the temperature T_max_ of pure silicone rubber (SR) and its composites before and after aging. An asterisk indicates the T_max_ value that differs from the others due to the addition of the ROKAmina surfactant.

From Figure 6, we can see that the SR/CLP/Fe/AM composite (6a) had the highest decomposition temperature (T_max_) after the aging process (Figure 6). This was due to the addition of the ROKAmina surfactant. There was an increase of approx. 40 °C in comparison with the pure SR sample, i.e., for the silicone rubber after the aging process. The addition of carbonyl iron to the composite (2) increased the decomposition temperature (T_max_) by approx. 1%. The aging process reduced the maximum decomposition temperature of the pure silicone rubber (SR) by approx. 2% (1a). The composite with added turmeric dissolved in oil (8) had a maximum decomposition temperature (T_max_) approx. 2% lower than the composite with turmeric powder added (4). The aging process resulted in a decrease in the maximum decomposition temperature (T_max_) of sample (8) by approx. 2% (8a), and of the sample (4) by approx. 1% (4a).

**Figure 7:**
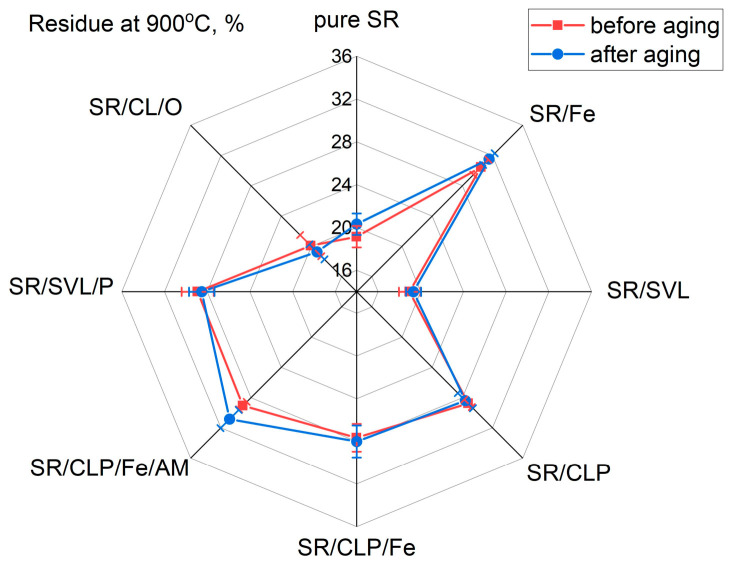
Comparison of the residue at 900 °C of pure SR and its composites with plant-fillers before and after aging.

Figure 7 shows that the largest residues after aging at a temperature of 900 °C were found in the case of composites containing carbonyl iron (2a, 5a, 6a). Composites with plant admixtures only (3a, 4a, 7a, 8a) had amounts of residues similar to those of the initial samples, i.e., pure silicone rubber, both before and after the aging process. The aging process generally resulted in an increase in residues after exposure to 900 °C (except for samples 7 and 8). The addition of the ROKAmina surfactant to the same composite, after the aging process, increased the residues after the exposure to temperature by more than 8% (sample 6a). The turmeric oil blend SR/CL/O composite left less residue than the turmeric powder blend at 900 °C, both before and after the aging process, by approx. 10% and approx. 7%, respectively.

### 3.3. Color Changes in the Polymer Composites Analysis

**Figure 8:**
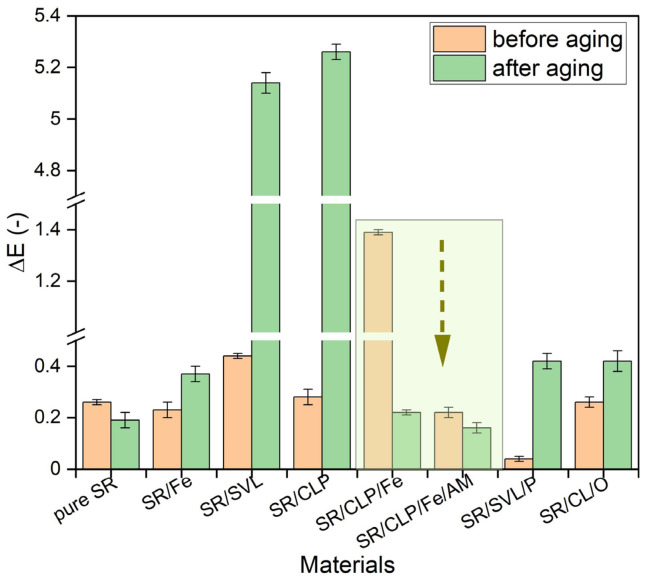
The impact of the aging process on the color change (ΔE) of silicone rubber and its composites. The shaded area indicates the greater color change (ΔE) before aging than after aging. The arrow indicates a decrease in the ΔE value in this area.

In the next stage of the research, changes in the color of the composites subjected to accelerated aging were determined. Changes in the color of the samples are usually the first sign of polymer degradation [43]. In subsequent studies, Figure 8 shows microscopic images of the color of the samples before and after exposure to light and water. The color change was based on the results of measurements of the color parameters L*, a*, b* according to the CIE Lab system, on the basis of which the color difference between the samples before and after aging was calculated [43]. Based on the obtained data, an absolute color difference was determined ΔE*. Table 3 presents the average parameter values L*, a*, b*, ΔE*, ΔL*, and ΔH*.

The results showed that the greatest difference in color after aging was observed in SR/SVL (3) and SR/CLP (4) composites; in the other cases, the color changes were insignificant. In general, the aged samples are more yellow in comparison to the reference samples, with the exception of samples 5a and 6a. These samples were modified with carbonyl iron, which turned the silicone rubber gray. For these samples, a reduction in (∆E*) to approximately 1.25 was observed. Such changes indicate that aging does not affect the color of the samples. The greatest changes in yellow color (∆E > 5) were recorded for composites 3 and 4 modified with plant powder.

The ΔE range in accordance with ISO 4892 [32] shows the following: 0 < ΔE < 1: imperceptible color changes; 1 < ΔE < 2: slight color changes, detectable only by an experienced observer; 2 < ΔE < 3.5: moderate color changes, detectable by an inexperienced observer; 3.5 < ΔE < 5: distinct color changes; ΔE > 5: significant color changes.

### 3.4. Microscopic Surface Analysis of the Silicone Rubber Composites

**Figure 9:**
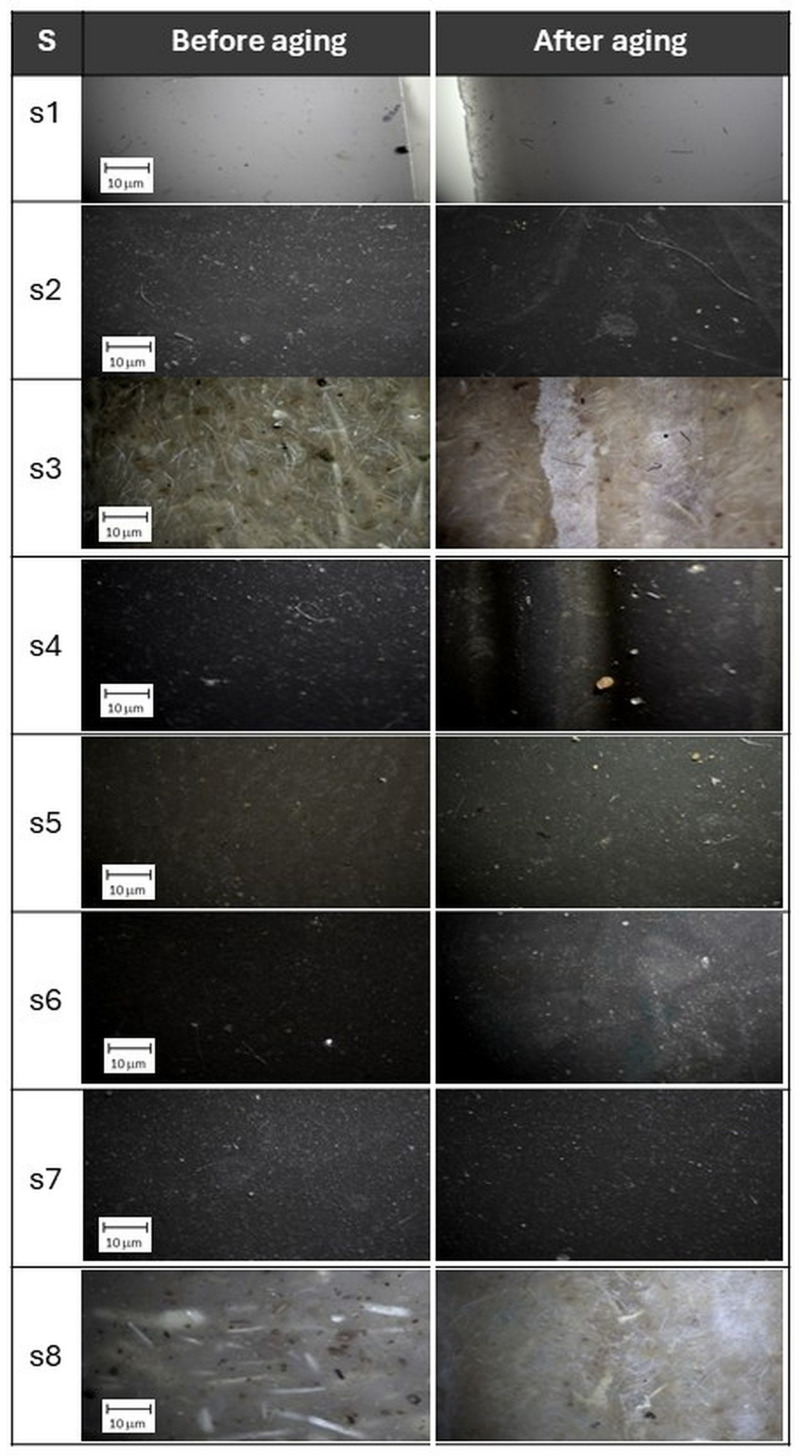
Microscopy pictures of the color changes in pure SR and its polymeric composites after the aging process.

Figure 9 shows microscopic images of the surface of silicone rubber (pure SR) samples before and after the aging process (right-hand column) for samples modified with turmeric, goldenrod, and carbonyl iron. The results of the microscopic observations correlate with the results of the colorimetric.

Microscopic observations of the samples (Figure 9) showed that the surfaces of the samples after aging were smooth, with no signs of cracks or delamination. Only the SR/CVL (S3), SR/CL/O (S8) composites, as well as sample pure silicone rubber (SR), exhibited a matting of the sample surfaces as a result of the interaction with water during the aging process. The results of microscopic observations showed that SR modified with carbonyl iron and plant-based fillers, such as curcuma and solidago, exhibits higher resistance to environmental factors, such as water and radiation, present during exposure to light sources, compared to pure silicone rubber.

### 3.5. FT-IR Analysis

**Figure 10:**
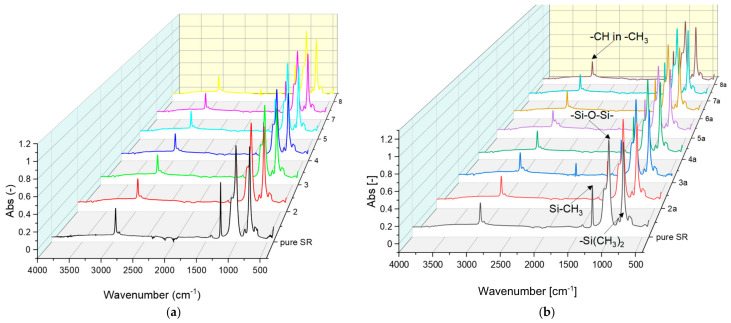
FTIR spectra of chemical bonds in pure SR and its composites: (**a**) before aging, (**b**) after aging process.

The chemical structure of silicone rubber modified by plant-based fillers and magnetic particles was verified by the FT-IR technique. FT-IR spectra of pure silicone rubber (pure SR) and SR composites, after exposure to the accelerated aging environments, are shown in Figure 10.

Spectroscopic analysis makes it possible to identify the chemical changes occurring in the main functional groups within silicone rubber as a result of chemical degradation [38]. As shown in Figure 10, the strongest absorption bands for the tested samples are between 900 cm^−1^ and 1300 cm^−1^; there are other weaker absorption bands at 786 cm^−1^, 1260 cm^−1^, and 2962 cm^−1^. The absorption band at 2960 cm^−1^ is assigned to the stretching vibration of –CH_3_, 1260 cm^−1^ is assigned to the bending vibration and rocking vibration of Si–CH_3,_ and the absorption band at 1006 cm^−1^ is assigned to the stretching vibration of the Si–O–Si bond on the backbone of silicone rubbers. The absorption band at 793 cm^−1^ is assigned to the coupling of the stretching vibration of Si–C and the rocking vibration of –CH_3_ [38].

Figure 10 presents the FTIR results of silicone rubber SR samples and their composites before and after the aging process. It shows that there are different changes in the heights of the absorption peaks of Si–O–Si (1006 cm^−1^) and Si–(CH_3_)_2_ (1260 cm^−1^) on the main chain in the silicone rubber. A comparative analysis of the Si-O-Si absorption peak height showed that, for most SR composites, the intensity of the Si-O-Si absorption peak (1006 cm^−1^) increased following aging, resulting in oxidative crosslinking. Only in the case of pure silicone rubber (SR) and composites SR/SVL (3a) and SR/CL/O (8a), modified plant-based fillers (*solidago and curcuma oil*) showed a decrease in the intensity of the absorption peak observed. The decrease in intensity of absorption bands suggested breaking of Si–O–Si or decomposition of methyl groups attached to silicon atoms [44]. As shown in Figure 10, the absorption peak intensity of Si-CH_3_ was reduced for silicone rubber, and composites SR/Fe (2a), SR/SVL (3a), and SR/CL/O (8a) after aging treatment. As a result, the reduction in the peak area of Si–(CH_3_)_2_ instead of Si–O–Si is selected to quantitatively characterize the degree of degradation during multi-factor aging. The results show that alternating temperature and water had a significant effect on the chemical degradation of the samples. This result reveals that the surface chemistry on the surfaces of silicone rubber samples did not change apparently in the testing environments, and the samples were stable under the aging. This can be attributed to the antioxidant properties of the fillers used. Goldenrod is an exceptionally rich source of polyphenols, giving it strong antioxidant properties; the antioxidants it contains, including flavonoids, neutralize free radicals [45,46]. The second filler added to the silicone matrix, such as turmeric, specifically curcumin, works in a similar way, as it contains phenolic and methoxyl groups that directly neutralize free radicals, which allows it to donate electrons to unstable oxygen molecules without losing its own stability [47].

## 4. Conclusions

The results of the study have shown that the use of accelerated aging techniques is of great importance for the systematic assessment of the degradation of polymer composites. The study also confirms the need to modify silicone rubber by adding natural fillers, such as goldenrod, turmeric, and iron carbonyl. The study analyzed changes in the material properties—namely Shore hardness, thermal properties, color, structure, and surface quality—of silicone rubber and its composites before and after an accelerated aging process. The samples were exposed to light from a xenon lamp, which emits white light similar to sunlight, in the presence of moisture, for a period of one year, in order to replicate the effects of natural atmospheric conditions. The bottom layer (layer B) of the two-layer composites consisted of silicone rubber, which was less elastic and contained no additives, whilst the top layer consisted of a mixture of silicone rubber, which was more elastic and contained 10% by mass of dried plants (*Solidago virgaurea* L. or *Curcuma longa* L.), and, in some composites, also magnetic particles in the form of iron carbonyl at 20% by mass.

The present work has led to the following conclusions:

− The hardness results show that all composite samples subjected to the aging process exhibit higher hardness compared to the non-aged samples. The greatest change was observed in the SR composite modified by a curcuma filler (SR/SVL and SR/SVL/P), whose hardness increased by approx. 30% compared to the non-aged silicone rubber (pure SR). Furthermore, the addition of plant-based fillers and carbonyl iron to the silicone rubber (SR) matrix led to an increase in the hardness of the SR composites (4, 5, 6, 8) of approx. 10%. Similarly, the addition of a filler—the amphoteric surfactant ROKAmina—increased the hardness of the composites (2, 5, 6) by approx. 9% compared with unmodified silicone rubber.
− The highest T_max_ value after aging was observed for the SR/Fe/AM composite containing carbonyl iron and the surfactant ROKAmina L30B. An increase of approx. 40 °C was recorded compared with unmodified silicone rubber (SR). The highest amounts of residue following exposure to 900 °C were observed for SR composites containing carbonyl iron.
− The results showed that the surface of the SR composites exhibited some changes in brightness and total color after aging. These promising results suggest that silicone rubber composites modified with iron- and amine-containing compounds are more resistant to aging than pure silicone rubber.
− The results of the microscopic observations showed that SR modified with carbonyl iron, plant-based fillers, such as curcuma and solidago, exhibit higher resistance to environmental factors, such as water and radiation, present during exposure to light sources, compared to pure silicone rubber.

## Figures and Tables

**Table 1:** List and composition (layer B) of the tested SR composites.

Sample	Polymer Composition
pure SR (1)	Silicone rubber Gumosil B
SR/Fe (2)	Silicone rubber Gumosil B + carbonyl iron (Fe)
SR/SVL (3)	Silicone rubber Gumosil B + *Solidago virgaurea* L. (dried from the field)
SR/CLP (4)	Silicone rubber Gumosil B + *Curcuma longa* L. (powder)
SR/CLP/Fe (5)	Silicone rubber Gumosil B + *Curcuma longa* L. + carbonyl iron (Fe)
SR/CLP/Fe/AM (6)	Silicone rubber Gumosil B + *Curcuma longa* L. + carbonyl iron (Fe) + ROKAmina L30B
SR/SVL/P (7)	Silicone rubber Gumosil B + *Solidago virgaurea* L. (from the pharmacy)
SR/CL/O (8)	Silicone rubber Gumosil B + *Curcuma longa* L. (oil)

**Table 2:** Characteristic thermogravimetric (TGA) data of pure silicone rubber (SR) and its composites before and after aging obtained in nitrogen.

Sample	T_5%_ [°C]	T_50%_ [°C]	Temperature for Peak Degradation T_max_ [°C]	Residue at 900 °C [%]
pure SR (1)	359.1	532.4	538.6	19.14
pure SR (1a)	361.0	519.3	530.1	20.32
SR/Fe (2)	378.1	544.3	544.3	30.45
SR/Fe (2a)	379.0	534.4	531.5	31.52
SR/SVL (3)	296.5	537.6	542.9	18.95
SR/SVL (3a)	293.6	530.9	538.0	19.32
SR/CLP (4)	295.7	541.1	538.7	28,76
SR/CLP (4a)	294.9	534.4	540.6	28.48
SR/CLP/Fe (5)	288.7	542.8	543.9	27.65
SR/CLP/Fe (5a)	293.5	540.4	540.6	28.00
SR/CLP/Fe/AM (6)	273.9	540.5	541.9	29.00
SR/CLP/Fe/AM (6a)	289.2	574.3	571.4	31.80
SR/SVL/P (7)	281.0	552.8	551.8	28.93
SR/SVL/P (7a)	281.1	544.9	543.3	28.51
SR/CL/O (8)	285.4	536.1	541.6	20.09
SR/CL/O (8a)	296.5	536.6	542.4	19.26

**Table 3:** Color parameters (L*, a*, b*, ΔE*, ΔL*, ΔH*) according to the CIE Lab system of pure SR and its composites before and after aging.

Sample	L* Value [-]	a* Value [-]	b* Value [-]	ΔE* [-]	ΔL* [-]	ΔH* [-]
Pure SR (1)	73.67	−1.88	−0.83	0.26	0.25	−0.01
Pure SR (1a)	74.44	−1.73	0.53	0.19	−0.18	0.05
SR/Fe (2)	23.61	0.30	0.15	0.23	−0.21	−0.06
SR/Fe (2a)	25.24	0.43	0.41	0.37	0.33	−0.13
SR/SVL (3)	48.69	4.68	25.64	0.44	−0.22	0.15
SR/SVL (3a)	51.35	10.65	17.44	5.14	−4.75	−1.32
SR/CLP (4)	48.69	4.68	25.64	0.28	0.27	0.15
SR/CLP (4a)	51.35	10.65	17.44	5.26	−0.57	−1.32
SR/CLP/Fe (5)	23.93	−0.50	12.32	1.39	−0.81	−0.31
SR/CLP/Fe (5a)	25.36	−0.43	0.86	0.22	−0.17	0.22
SR/CLP/Fe/AM (6)	22.77	0.56	8.26	0.22	−0.16	−0.01
SR/CLP/Fe/AM (6a)	26.93	−1.15	1.55	0.16	0.13	0.04
SR/SVL/P (7)	20.30	3.13	8.06	0.04	−0.02	0.03
SR/SVL/P (7a)	19.81	3.15	4.60	0.42	0.29	0.14
SR/CL/O (8)	41.97	7.32	20.15	0.26	−0.09	−0.06
SR/CL/O (8a)	52.86	12.19	23.19	0.42	0.31	−0.17

## Data Availability

The original contributions presented in this study are included in the article. Further inquiries can be directed to the corresponding author.

## Abbreviations

The following abbreviations are used in this manuscript:

SR	silicone rubber
PP	polypropylene
PP/CB	polypropylene/carbon soot (PP/CB)
PU	polyurethane
SR/SVL	silicone rubber Gumosil B + carbonyl iron (Fe)
SR/CLP	silicone rubber Gumosil B + *Solidago virgaurea* L. (dried from the field)
SR/CLP/Fe	silicone rubber Gumosil B + *Curcuma longa* L. + carbonyl iron (Fe)
SR/CLP/Fe/AM	silicone rubber Gumosil B + *Curcuma longa* L. + carbonyl iron (Fe) + ROKAmina L30B
SR/SVL/P	silicone rubber Gumosil B + *Solidago virgaurea* L. (from the pharmacy)
SR/CL/O	silicone rubber Gumosil B + *Curcuma longa* L. (oil)
HTV	High Temperature Vulcanized

## References

[1] Shit S.C., Shah P. (2013). A Review on Silicone Rubber. Natl. Acad. Sci. Lett..

[2] Kaneko T., Ito S., Minakawa T., Hirai N., Ohki Y. (2019). Degradation Mechanisms of Silicone Rubber under Different Aging Conditions. Polym. Degrad. Stab..

[3] Masson J.-F., Lopez–Carreon I., Wu J., Obukohwo O., Collins P., Riahinezhad M., Esmizadeh E. (2022). Degradation and Service-Life Prediction of Silicone Rubber in a Highly Alkaline Environment Simulating Concrete. Eng. Fail. Anal..

[4] Wu J., Dong J., Wang Y., Gond B.K. (2017). Thermal Oxidation Ageing Effects on Silicone Rubber Sealing Performance. Polym. Degrad. Stab..

[5] Cosnita M., Cazan C., Pop M.A., Cristea D. (2023). Aging Resistance under Short Time Ultraviolet (UV) Radiations of Polymer Wood Composites Entirely Based on Wastes. Environ. Technol. Innov..

[6] Krauklis A.E., Karl C.W., Rocha I.B.C.M., Burlakovs J., Ozola-Davidane R., Gagani A.I., Starkova O. (2022). Modelling of Environmental Ageing of Polymers and Polymer Composites—Modular and Multiscale Methods. Polymers.

[7] Song Y., Deng J., Xu Z., Nie Y., Lan Z. (2022). Effect of Thermal Aging on Mechanical Properties and Color Difference of Glass Fiber/Polyetherimide (GF/PEI) Composites. Polymers.

[8] Zubair Z., Razzaq W., Abbas A., Hussain F., Ghalib E., Ayyoob M., Hamdani S.T.A. (2025). Thermal Activation and Dynamic Mechanical Characterization of Glass-Reinforced Shape Memory Polymer Composites for Medical Applications. Text. Res. J..

[9] Garbacz Ł., Klepka T., Longwic F. (2021). The Influence of the Aging Process on the Change of Selected Strength Properties of Polypropylene Compositions with Mineral Fillers. Adv. Sci. Technol. Res. J..

[10] Czarnecka-Komorowska D., Chandra S., Kopeć B., Borowski J., Garbacz T. (2022). Investigating the Effect of Photo-Oxidative Degradation on the Ageing Resistance of the Car Mudflaps Manufactured with Post-Production High-Density Polyethylene Wastes. Adv. Sci. Technol. Res. J..

[11] Samujło B. (2020). The Effect of the Aging Process on Selected Properties of Polypropylene Modified by Natural Fillers. Adv. Sci. Technol. Res. J..

[12] Busiak R., Masek A., Węgier A., Rylski A. (2022). Accelerated Aging of Epoxy Biocomposites Filled with Cellulose. Materials.

[13] Boubakri A., Haddar N., Elleuch K., Bienvenu Y. (2011). Influence of Thermal Aging on Tensile and Creep Behavior of Thermoplastic Polyurethane. Comptes Rendus Mécanique.

[14] Magiera A., Kuźnia M., Jerzak W. (2025). Analysis of the Structural, Chemical, and Mechanical Characteristics of Polyurethane Foam Infused with Waste from Thermal Processing. Materials.

[15] Feng J., Zhang Q., Tu Z., Tu W., Wan Z., Pan M., Zhang H. (2014). Degradation of Silicone Rubbers with Different Hardness in Various Aqueous Solutions. Polym. Degrad. Stab..

[16] Li G., Tan J., Gong J. (2012). Degradation of the Elastomeric Gasket Material in a Simulated and Four Accelerated Proton Exchange Membrane Fuel Cell Environments. J. Power Sources.

[17] Sanchez-Sobrado O., Visniakov N., Bureika G., Losada R., Rodriguez E. (2024). Effect of the Chemical Surrounding Environment on the Physical and Mechanical Properties of Aged Thermoplastic Polymers. Heliyon.

[18] The Influence of Accelerated Aging on Selected Mechanical Properties of Polypropylene with Organic Fillers. https://www.researchgate.net/publication/363670867_The_influence_of_accelerated_aging_on_selected_mechanical_properties_of_polypropylene_with_organic_fillers.

[19] Hong S.K. (2021). The Effect of Long Term Hygrothermal Aging by Immersion on Carbon/Epoxy Composites Exposed to the Heat Sources for Naval and Marine Applications. IOP Conf. Ser. Mater. Sci. Eng..

[20] Bouregba R., Abbes B., Bouziane M., Bellali M., Salem M. (2024). Effect of Thermal Ageing on the Thermal and Mechanical Properties of Polypropylene and Polypropylene Micro-Composites. J. Polym. Mater..

[21] Susanto T., Affandy R., Katon G., Rahmaniar (2018). Thermal Aging Properties of Natural Rubber-Styrene Butadiene Rubber Composites Filled with Modified Starch from Dioscorea Hispida Denst Extract Prepared by Latex Compounding Method. AIP Conf. Proc..

[22] Szatkowski P., Gralewski J., Suchorowiec K., Kosowska K., Mielan B., Kisilewicz M. (2024). Aging Process of Biocomposites with the PLA Matrix Modified with Different Types of Cellulose. Materials.

[23] Bendjaouahdou C., Bensaad S. (2018). Aging Studies of a Polypropylene and Natural Rubber Blend. Int. J. Ind. Chem..

[24] Perales-Martínez I.A., Palacios-Pineda L.M., Lozano-Sánchez L.M., Martínez-Romero O., Puente-Cordova J.G., Elías-Zúñiga A. (2017). Enhancement of a Magnetorheological PDMS Elastomer with Carbonyl Iron Particles. Polym. Test..

[25] Miękoś E., Cichomski M., Zieliński M., Klepka T., Sroczyński D., Fenyk A. (2021). Modification of the Properties of Polymer Composites in a Constant Magnetic Field Environment. Materials.

[26] Tian W.-W., Liu L., Chen P., Yu D.-M., Li Q.-M., Hua H., Zhao J.-N. (2025). Curcuma Longa (Turmeric): From Traditional Applications to Modern Plant Medicine Research Hotspots. Chin. Med..

[27] Xu J., Zhang Y., Feng Y.B., Qiu T., Wang G., Liu R. (2018). Electromagnetic and Mechanical Properties of Carbonyl Iron Powder-Filled Methyl Vinyl Silicone Rubber during Thermal Aging. Polym. Compos..

[28] Abshinova M.A., Kuřitka I., Kazantseva N.E., Vilčáková J., Sáha P. (2009). Thermomagnetic Stability and Heat-Resistance Properties of Carbonyl Iron Filled Siloxanes. Mater. Chem. Phys..

[29] Englert M., Minister F., Moussaoui A., Pisula W. (2022). Mechanical Properties of Thermo-Oxidative Aged Silicone Rubber Thermally Stabilized by Titanium Oxide Based Fillers. Polym. Test..

[30] Silikony Polskie. https://www.sklep.silikonypolskie.pl/.

[31] (2021). Admin ROKAmina L30B MB-Lauryl Betaine. PCC Group. https://www.products.pcc.eu/en/id/1310009/rokamina-l30b-mb-lauryl-betaine-3/.

[32] (2013). Plastics—Methods of Exposure to Laboratory Light Sources—Part 2: Xenon-Arc Lamps.

[33] (2003). Plastics and Ebonite—Determination of Indentation Hardness by Means of a Durometer (Shore Hardness).

[34] (2022). Plastics—Thermogravimetry (TG) of Polymers. Part 1: General Principles.

[35] (2025). Plastics—Determination of Changes in Colour and Variations in Properties After Exposure to Glass-Filtered Solar Radiation, Natural Weathering or Laboratory Radiation Sources.

[36] Technical Reports|CIE. https://www.cie.co.at/publications/technical-reports.

[37] Chorobiński M., Skowroński Ł., Bieliński M. (2019). Metodyka wyznaczania wybranych charakterystyk barwienia polietylenu z wykorzystaniem systemu CIELab. Polimery.

[38] Wu F., Chen B., Yan Y., Chen Y., Pan M. (2018). Degradation of Silicone Rubbers as Sealing Materials for Proton Exchange Membrane Fuel Cells under Temperature Cycling. Polymers.

[39] Delor-Jestin F., Tomer N.S., Singh R.P., Lacoste J. (2006). Characterization of Polydimethylsiloxane Rubber upon Photochemical, Thermal, Salt-Fog Ageings and Exposure to Acid Vapours. e-Polymers.

[40] Zeng S., Li W., Peng Y., Zhang Y., Zhang G. (2023). Mechanism of Accelerated Deterioration of High-Temperature Vulcanized Silicone Rubber under Multi-Factor Aging Tests Considering Temperature Cycling. Polymers.

[41] Han R., Li Y., Zhu Q., Niu K. (2022). Research on the Preparation and Thermal Stability of Silicone Rubber Composites: A Review. Compos. Part C Open Access.

[42] El-Sayed S.A., Khass T.M., Mostafa M.E. (2024). Thermal Degradation Behaviour and Chemical Kinetic Characteristics of Biomass Pyrolysis Using TG/DTG/DTA Techniques. Biomass Convers. Biorefin..

[43] Malicka A., Rułka K., Latos-Brozio M., Masek A. (2024). Elastomeric Compositions of Ethylene–Norbornene Copolymer Containing Biofillers Based on Coffee and Tea Waste. Materials.

[44] Wang R.-Y., Dou Z.-F., Li H.-S., Li N., Liu X.-R., Zhang W.-F. (2025). Degradation Behavior and Aging Mechanisms of Silicone Rubber under Ultraviolet–Thermal–Humidity Coupling in Simulated Tropical Marine Atmospheric Environment. Polymer.

[45] Piątkowska E., Biel W., Witkowicz R., Kępińska-Pacelik J. (2022). Chemical Composition and Antioxidant Activity of Asteraceae Family Plants. Appl. Sci..

[46] Katalinic V., Milos M., Kulisic T., Jukic M. (2006). Screening of 70 Medicinal Plant Extracts for Antioxidant Capacity and Total Phenols. Food Chem..

[47] Feng J.-Y., Liu Z.-Q. (2009). Phenolic and Enolic Hydroxyl Groups in Curcumin: Which Plays the Major Role in Scavenging Radicals?. J. Agric. Food Chem..

